# Continuous deep brain stimulation of the nucleus accumbens reduces food intake but does not affect body weight in mice fed a high-fat diet

**DOI:** 10.1038/s41598-023-45511-7

**Published:** 2023-11-02

**Authors:** Harold F. Hounchonou, Hui Tang, Raik Paulat, Andrea Kühn, Joachim Spranger, Christoph van Riesen, Lukas Maurer

**Affiliations:** 1 Department of Endocrinology and Metabolism, Charité University Medicine Berlin, Berlin, Germany; 2 Max Rubner Center for Cardiovascular Metabolic Renal Research, Charité University Medicine Berlin, Berlin, Germany; 3 Department of Neurosurgery, Hannover Medical School, Hannover, Germany; 4 Movement Disorder and Neuromodulation Unit, Department of Neurology, Charité University Medicine Berlin, Berlin, Germany; 5 Department of Neurology, University Medical Center Göttingen, Göttingen, Germany

**Keywords:** Obesity, Reward

## Abstract

Obesity is an enormous health problem, and many patients do not respond to any of the available therapies. Deep brain stimulation (DBS) is currently investigated as a potential treatment for morbid obesity. In this study, we tested the hypothesis that high-frequency DBS targeting the nucleus accumbens (NAc) shell region reduces food intake and weight gain in mice fed a high-fat diet. We implanted male C57BL/6J mice with bilateral electrodes and a head-mounted microstimulator enabling continuous stimulation for up to 5 weeks. In successfully operated animals (n = 9 per group, high-frequency vs. sham stimulation), we investigated immediate and long-term stimulation effects on metabolic and behavioral phenotypes. Here we show that stimulation acutely induced a transient reduction in energy expenditure and locomotor activity but did not significantly affect spontaneous food intake, social interaction, anxiety or exploratory behaviors. In contrast, continuous stimulation over 5 weeks led to a decrease in food intake and thigmotaxis (the tendency to stay near walls in an open lit arena). However, chronic stimulation did not substantially change weight gain in mice fed a high-fat diet. Our results do not support the use of continuous high-frequency NAc shell DBS as a treatment for obesity. However, DBS can alter obesity-related parameters with differing short and long-term effects. Therefore, future research should employ time and context-sensitive experimental designs to assess the potential of DBS for clinical translation in this area.

## Introduction

Obesity is an enormous health burden affecting over 600 million people worldwide, and many patients fail to respond to available treatments^1^. Even after bariatric surgery, patients suffer from obesity and weight regain, especially in the long run^2^. There is an enormous need for additional therapeutic approaches, especially in morbid, therapy-refractory obesity. The potential of DBS as a treatment for advanced, medication-resistant movement and, to some extent, neuropsychiatric disorders sparked great interest in exploring DBS as a treatment for severe obesity^3^.

So far, researchers have mainly explored two DBS targets to treat obesity: (i) stimulation of hypothalamic centers was used as an attempt to modulate energy homeostasis^4–6^, and (ii) the nucleus accumbens was stimulated to manipulate reward-related aspects of food consumption^7–9^. Although some promising human data on NAc DBS in obesity have emerged recently, the results are limited to a small number of case reports^7–10^. Meanwhile, successful implementation in well-controlled clinical trials is lacking^11^.

Multiple studies in rodents evaluated the acute effects of NAc DBS on food intake and body weight with inconsistent results. High-frequency stimulation of the NAc has been shown to increase^12^, decrease^13^, or have no effect^14,15^ on food intake in rodent obesity models. With a more specific emphasis on the reward-related aspect of food consumption, NAc DBS has been investigated in rodent binge-eating models; where DBS induced a significant reduction in binge eating^16,17^. Interestingly, the effect of a prolonged application on food consumption seemed to decline over time^17,18^. Given the scarcity of data in preclinical models using chronic stimulation paradigms, there is great uncertainty with respect to potential long-term DBS effects.

To characterize acute and chronic effects of NAc DBS, we implanted a wireless DBS system and performed a continuous, long-term (up to 5 weeks) stimulation without movement restrictions in mice fed a high-fat diet. We intended to assess the stability of a presumed effect and to detect potential modulatory DBS effects occurring only after prolonged stimulation^19,20^.

A general problem in this research area is to evaluate the specificity of the presumably “negative” impact of DBS on food intake and body weight. Stress-related unspecific alterations due to the experimental procedure can have an enormous impact, especially concerning metabolic endpoints^21,22^. NAc DBS can induce anxiolytic effects in the context of neuropsychiatric disorders in rodents and humans^23,24^. At the same time, stimulation induced side effects in terms of increased anxiety, agitation and hypomania have been documented^25^. Thus, there is an obvious potential interplay between these factors and changes in appetite and body weight^22^.

Therefore, we tried to implement a continuous stimulation phase with minimal animal handling and to combine comprehensive metabolic phenotyping using an indirect gas calorimetry system with a behavioral characterization in the open field arena.

We intended to test two main hypothesis: (i) continuous high-frequency DBS targeting the NAc shell induces a reduction in food intake^12,17,26^, and (ii) continuous high-frequency DBS reduces weight gain in mice fed a high-fat diet^19,27^.

Additionally, we performed an exploratory analysis on potential acute and chronic side effects of the stimulation with respect to energy expenditure, locomotor activity and anxiety-like behaviors (social interaction or thigmotactic ratio).

A predominant limitation for the clinical application of DBS as a therapeutic approach in obesity, is the enormous uncertainty regarding the optimal target region^13,27,28^, the stimulation paradigm^29–31^ and the specific clinical context^4,7–10^. While this challenge also applies for the preclinical exploration, animal models offer the opportunity to evaluate individual paradigms beforehand.

In order to expand the preclinical foundation for a potential clinical translation, we focused on the continuous high-frequency paradigm as the most used DBS application in clinical practice. We selected the NAc as central target within the brain reward circuit and the context of a diet-induced obesity model to resemble ubiquitous energy-dense food availability as one of the key features in obesity. We focused on the HFD-fed C57BL/6J mouse model, since it resembles the main obesity associated features of impaired glucose tolerance^32^, leptin resistance^33^ as well as behavioral and neuroimmunological changes^34^.

Our findings indicate divergent acute and long-term effects of NAc DBS on food intake, energy expenditure, and anxiety-related behavior. Starting the stimulation induced a transient reduction in energy expenditure and spontaneous locomotor activity but not ad libitum food intake, social interaction or anxiety, and exploratory behaviors. Only after 5 weeks of continuous stimulation, we detected a decrease in food intake and thigmotaxis, but DBS did not prevent weight gain.

## Results

### Cohort description

The animals were grouped according to their body weight before the first activation of the DBS system to allow a similar body weight distribution in the two groups (bodyweight at baseline: 25.96 ± 0.85 g (SHAM) vs. 25.64 ± 0.88 g (HFS); p = 0.8).

A final 18 of 24 animals were included in the data analysis. Six animals were excluded due to failed or unstable device implantation (n = 2), incorrect electrode position (n = 2), or stimulator malfunction (n = 2). As a result, nine high-frequency stimulated (HFS) mice were compared with nine implanted but unstimulated (SHAM) mice in the final analysis.

**Figure 1:**
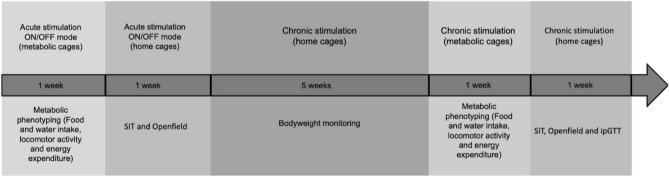
Experimental timeline.

The experimental timeline is depicted in Fig. 1.

### Body weight

**Figure 2:**
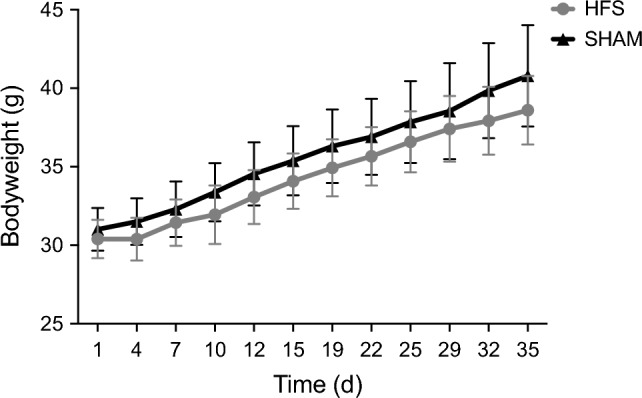
Development of absolute body weight in both groups over 5 weeks of continuous stimulation.

Body weight development in both groups during the chronic stimulation phase is illustrated in Fig. 2. After chronic stimulation, the mean bodyweight was 38.6 ± 2.19 g in the HFS group and 40.8 ± 3.23 g in the SHAM group. Continuous DBS for 5 weeks did not significantly reduce weight gain in mice on a high-fat diet (34.4 ± 0.85 g (HFS) vs. 35.7 ± 0.94 g (SHAM); p = 0.98).

### Food intake

The first two food intake measurements were performed in metabolic cages during acute stimulation days (d1 and d3). We compared the difference in cumulative food intake after switching between stimulation-on and stimulation-off on these 2 days. Animals were handled in parallel but only in the HFS group the stimulator was activated. The results showed no significant difference within the HFS group during acute stimulation (5.63 ± 0.18 g (ON) vs. 5.78 ± 0.24 g (OFF); p = 0.15) or between the two groups over the same period (5.56 ± 0.40 g (SHAM) vs. 5.63 ± 0.18 g (HFS); p = 0.44).

**Figure 3:**
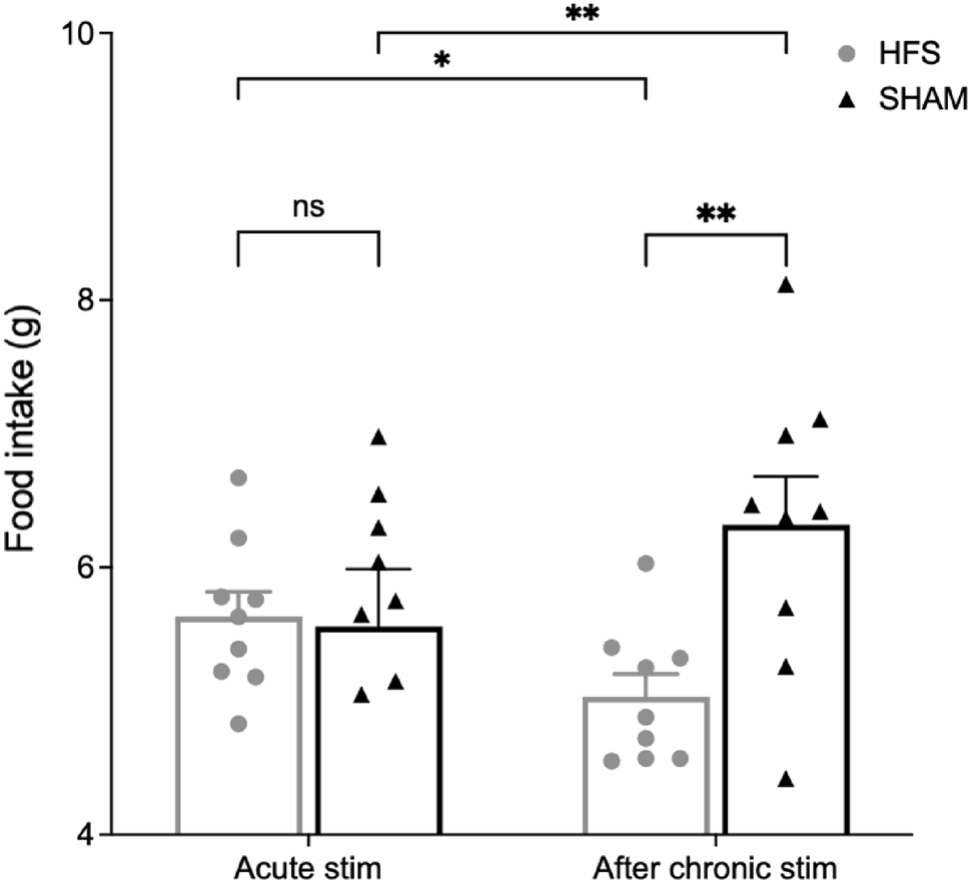
Cumulative food intake in both groups during acute stimulation and after chronic stimulation. Cumulative food intake on acute stimulation days showed no significant difference between both groups (*ns* no significance; p = 0.16). However, after chronic stimulation, cumulative food intake on corresponding days was significantly lower in the HFS group compared to the SHAM group (**p = 0.002).

After 5 weeks of chronic stimulation, cumulative food intake over the 4-day measurement period was significantly lower in the stimulation group than in the control group (12.69 ± 0.60 g (SHAM) vs. 10.50 ± 0.52 g (HFS); p = 0.009). To allow for a quantitative comparison to the initial measurement, we additionally analyzed food intake on the corresponding days (d1′ and d3′ active stimulation during the first session). The results indicate a significantly higher food intake in the sham group compared to the HFS group (6.32 ± 0.34 g (SHAM) vs. 5.03 ± 0.16 g (HFS); p = 0.002). Food intake data are summarized in Fig. 3, showing a between-group difference after chronic stimulation, which was not present in the initial acute stimulation period.

Unfortunately, our attempt to track food intake in the home cages during stimulation was not successful due to numerous food pellets being “spilled” by the mice, making accurate quantification impossible.

### Water intake

No concomitant effect on water intake was detected in the metabolic cages. No significant difference was observed in mean water intake between the two groups either during acute stimulation (5.05 ± 0.32 ml (SHAM) vs. 4.64 ± 0.17 ml (HFS); p = 0.15) or after chronic stimulation (4.92 ± 0.32 ml (SHAM) vs. 4.35 ± 0.14 (HFS); p = 0.08).

### Energy expenditure and spontaneous locomotor activity

**Figure 4:**
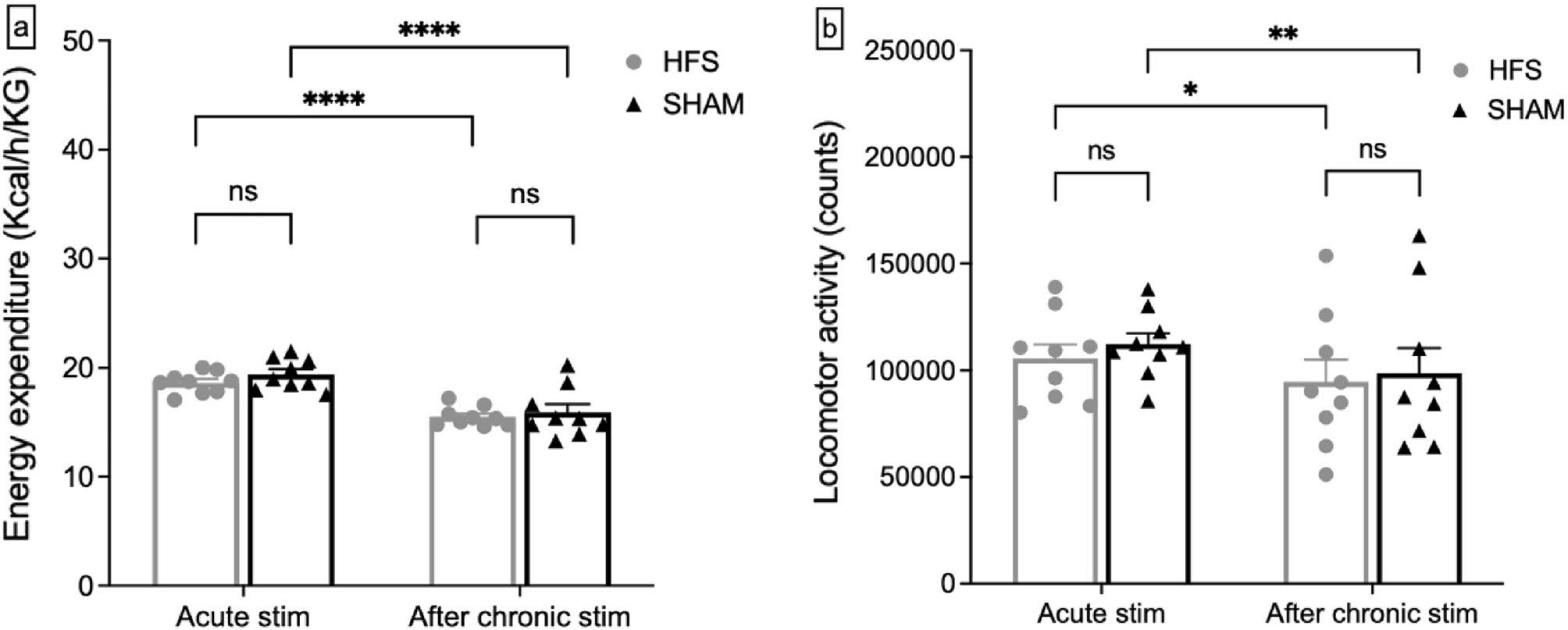
(**a**) Mean energy expenditure in both groups on acute stimulation days and after chronic stimulation. No significant effect was detected in overall energy expenditure either during acute stimulation (*ns* no significance; p = 0.09) or after chronic stimulation (p = 0.31). (**b**) Cumulative locomotor activity during acute stimulation and after chronic stimulation in both groups. Stimulation produced no significant effect either during acute stimulation (*ns* no significance; p = 0.21) or after the chronic stimulation phase (p = 0.40).

Overall energy expenditure and spontaneous locomotor activity measured on day 1 and day 3 during initiation of the HFS stimulation showed no significant differences (energy expenditure: 19.42 ± 0.44 kcal/h/KG (SHAM) vs. 18.64 ± 0.31 kcal/h/KG (HFS), p = 0.09; locomotor activity: 112,337.78 ± 4891.43 counts (SHAM) vs. 105,474.67 ± 6453.18 counts (HFS), p = 0.21). The stimulation did also not produce a detectable effect after 5 weeks of continuous stimulation (energy expenditure: 15.91 ± 0.71 kcal/h/KG (SHAM) vs. 15.51 ± 0.28 kcal/h/KG (HFS), p = 0.31; locomotor activity: 98,629.89 ± 11,221.41 counts (SHAM) vs. 94,595.33 ± 9838.97 counts (HFS), p = 0.40). These results are summarized in Fig. 4.

**Figure 5:**
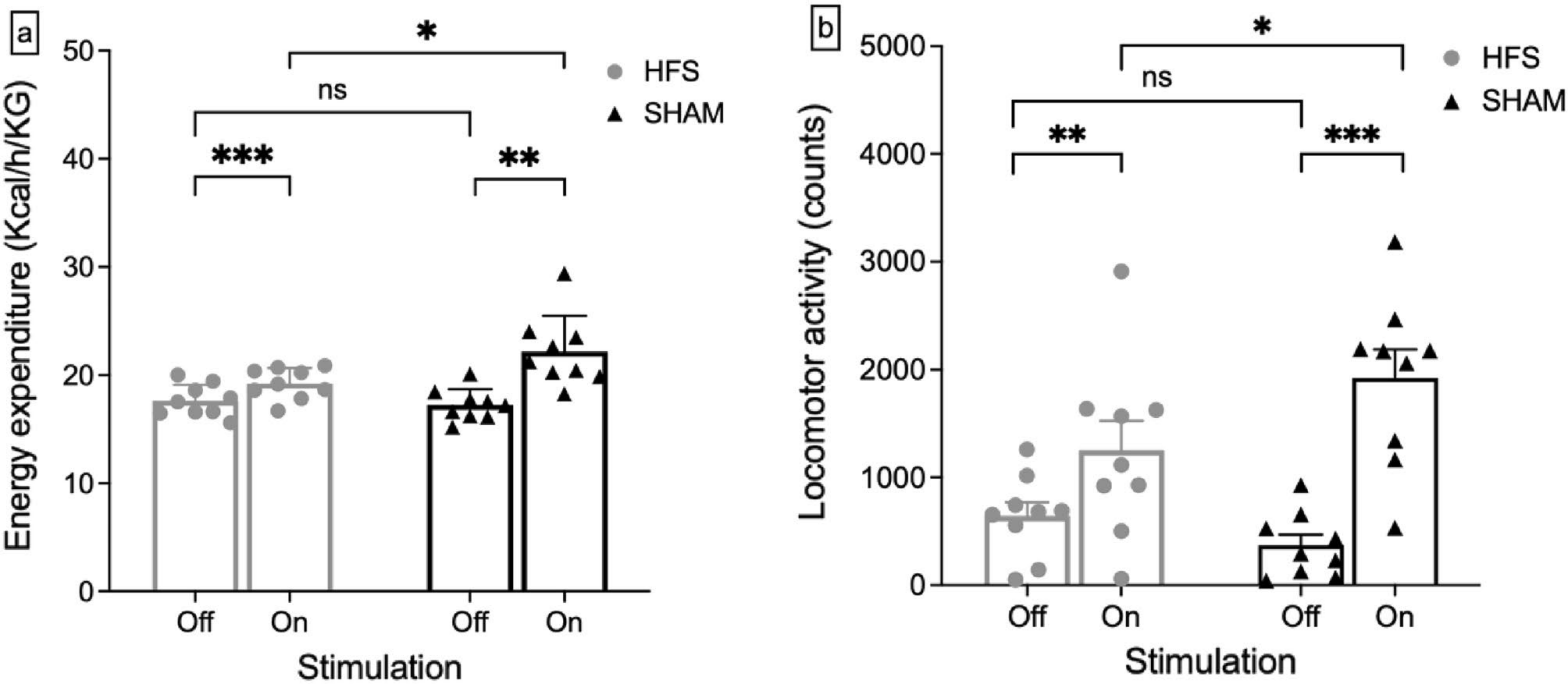
(**a**) Energy expenditure directly before and after switching stimulation on [± 30 min] on acute stimulation days. Directly after switching-on, energy expenditure was significantly lower in the HFS group compared to the sham group (*p < 0.05). (**b**) Locomotor activity directly before and after switching stimulation on [± 30 min] on acute stimulation days. Directly after switching-on, locomotor activity was significantly lower in the HFS group compared to the sham group (*p < 0.05).

In the acute stimulation setting immediately after switching on the DBS system [± 30 min on day 1 and day 3], mice in the HFS group showed lower energy expenditure than the sham group (Stim OFF: 17.27 ± 0.46 kcal/h/KG (SHAM) vs. 17.65 ± 0.46 kcal/h/KG (HFS); Stim ON: 22.19 ± 1.02 kcal/h/KG (SHAM) vs. 19.25 ± 0.45 kcal/h/KG (HFS); p = 0.004, see Fig. 5a).

A similar effect was found on locomotor activity (Stim OFF: 368.22 ± 93.12 counts (SHAM) vs. 643 ± 118.56 counts (HFS); Stim ON: 1921.22 ± 741.14 counts vs. 1252 ± 771.15 counts; p = 0.01, see Fig. 5b).

However, this effect on energy expenditure and locomotor rapidly diminished afterwards and showed no overall impact on the total energy expenditure per day.

### Social-interaction-test

**Figure 6:**
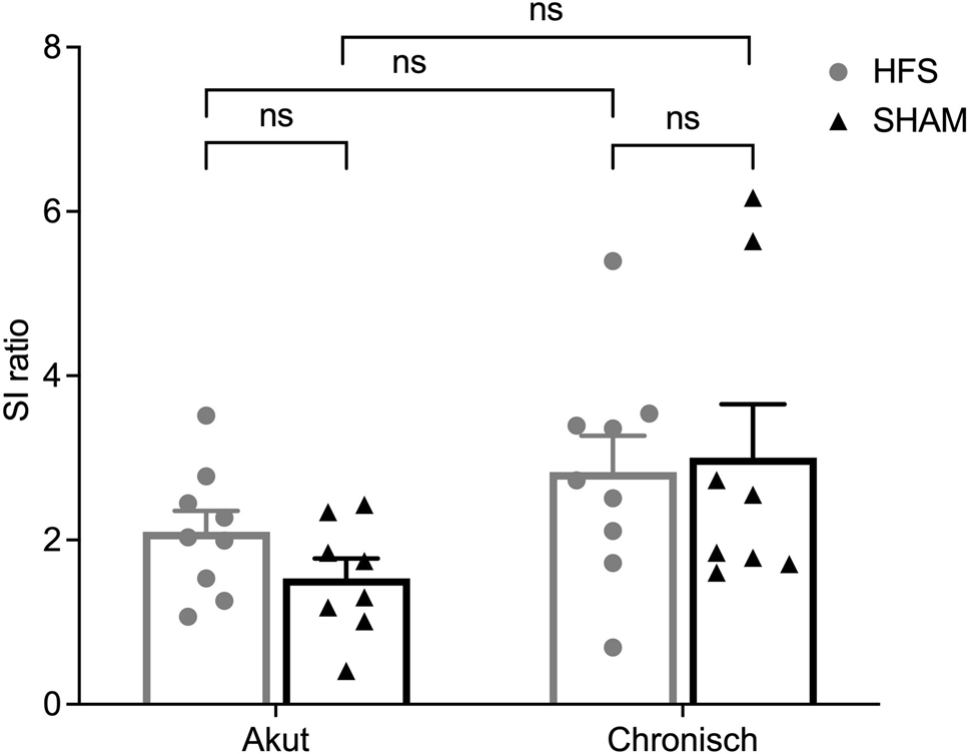
Social interaction (SI) ratio of HFS and sham group animals during acute stimulation and after chronic stimulation. No significant difference was found during acute stimulation (*ns* non-significant; p = 0.06) and after chronic stimulation (*ns* non-significant, p = 0.41).

Analysis of the social interaction ratio (SI ratio) is depicted in Fig. 6 and revealed no significant difference between the two groups, neither during acute stimulation (1.53 ± 0.24 (SHAM) vs. 2.1 ± 0.26 (HFS); p = 0.06) nor after 5 weeks of chronic stimulation (3.00 ± 0.65 (SHAM) vs. 2.83 ± 0.44 (HFS); p = 0.41).

### Open-field-test

**Figure 7:**
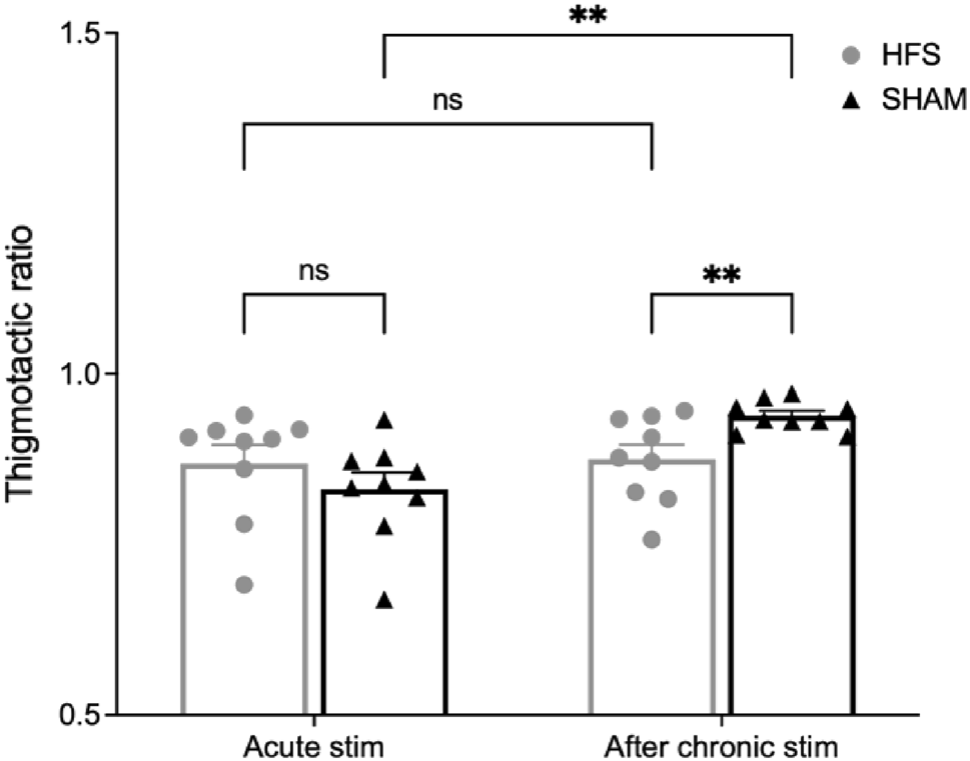
Comparison of thigmotactic ratio during acute and chronic stimulation in both groups. Chronic stimulation resulted in a significantly higher thigmotactic ratio in the sham group compared to the HFS group (**p < 0.01).

To analyze the potential effects of DBS on anxiety and exploratory behavior, we performed a 30 min open field test looking at the overall motor activity and thigmotaxis. The overall locomotor activity did not show significant differences with respect to average movement velocity or total distance traveled during acute or at the end of the chronic stimulation period (data not shown). In addition, the thigmotactic ratio did not differ on acute stimulation (0.83 ± 0.02 (SHAM) vs. 0.87 ± 0.03 (HFS); p = 0.16). However, after 5 weeks of continuous stimulation, the thigmotactic ratio was higher in the sham group than in the HFS group (0.94 ± 0.01 (SHAM) vs. 0.88 ± 0.02 (HFS); p = 0.01). Furthermore, the within-group comparison showed a significant increase in the sham group (p = 0.004), while no difference was observed in the HFS group (p = 0.839). The results are illustrated in Fig. 7.

### Intraperitoneal glucose-tolerance-test (ipGTT)

**Figure 8:**
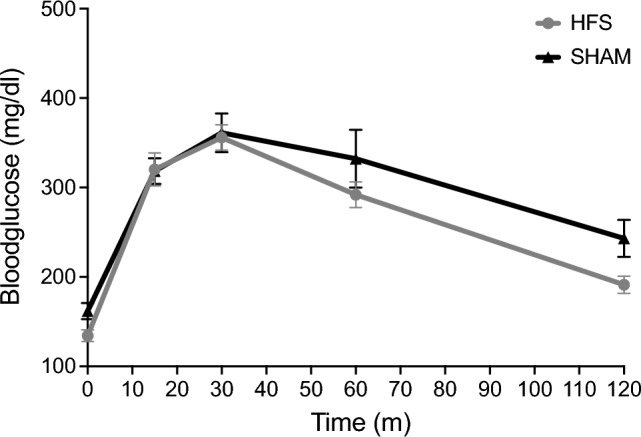
Blood glucose development during i.p. glucose tolerance test. No overall significant difference was found between both groups (p = 0.14).

Fasting glucose levels were similar in both groups and showed a similar peak and overall non-differential AUC. There was no significant overall difference between both groups (283.3 ± 36.1 mg/dl (SHAM) vs. 258.7 ± 41.2 mg/dl (HFS), p = 0.14, see Fig. 8). Only two hours after the injection mice on HFS showed a significantly lower glucose level (243.1 ± 20.71 (SHAM) vs. 191.2 ± 9.6 (HFS); p = 0.03).

## Discussion

Overall, continuous high-frequency stimulation of the NAc over 5 weeks did not reduce weight gain in mice fed a high-fat diet. Therefore, within the limitations of the experimental design, we do not confirm the hypothesis that NAc DBS can induce a stable reduction in food intake and body weight gain.

Despite that, we show that NAc shell DBS affects food- and anxiety-related behaviors and physical activity that differ between acute and chronic stimulation.

The initial activation of the DBS system induced a transient reduction in energy expenditure and spontaneous locomotor activity measured within the metabolic chamber. In contrast, it did not immediately alter exploratory behavior in the open field test. After taking them out of the cage and handling them (necessary for activation of the microstimulator but performed in parallel in both groups), all animals showed an elevated locomotor activity and concomitant increase in energy expenditure. This happened in both groups irrespective of an active stimulation presumably as consequence of activation and stress due to human interference. In comparison to the “SHAM” group, stimulated animals showed a significantly ameliorated response. As any handling would stress the animal^35^, this might indicate a blunted response to the stress associated with the stimulator activation procedure. The role of NAc DBS in normalizing behavioral and humoral stress responses in a mouse model of chronic mild stress has recently been demonstrated^36^. However, despite continued stimulation, the effect seemed to wear off rapidly and did not translate to an altered 24-h energy expenditure or locomotor activity. The rapid alignment could be due to a dependency on a preceding stressor or the wearing-off effects of the DBS itself. A rapidly decrementing stimulation effect has been described in rodent binge-eating models^17,18^. Furthermore, in our experiment, the relative decrease in locomotor activity in the metabolic chamber within the first 30 min after DBS activation does not translate into a parallel effect on anxiety or exploratory behavior measured in the open field test. This result would be in line with the data indicating that the effects of NAc DBS on anxiety and exploratory behaviors seem to normalize model-induced impairments rather than promote these behaviors in general^36,37^.

In contrast to the acute setting, continuous stimulation over 5 weeks reduced thigmotaxis (the tendency to stay near walls in an open lit arena) without altering locomotor activity. The thigmotactic ratio (distance traveled close to the walls divided by the total travel distance) is an established marker for anxiety-related behavior in mice and has been shown to respond to chronic intermittent DBS in rodent depression models^38,39^. Our results repeat those findings in an obesity mouse model. After prolonged high fat diet exposure, the control group showed the expected weight gain, an increase in HFD consumption^40^ and a higher thigmotactic ratio. It is important to note that these changes developed over time irrespective of active DBS. As animals grow and gain weight their daily energy intake increases^41^. Prolonged HFD feeding itself seems to produces anxiety-like behavior in mice potentially via influencing the serotonin system^42^. This highlights the importance of evaluating every potential DBS effect in relation to the experimental context it has been applied to. The fact that chronic stimulation ameliorated the development of this behavioral change, underscores the need for longer-term DBS application to detect effects, especially concerning neuropsychiatric conditions. Similarly, the reduction of food intake only occurred after prolonged stimulation. Given the solitary manifestation of the decreased consumption without concomitant behavioral impairment, it seems unlikely that a stress or discomfort-related alteration due to the experimental procedure is the predominant cause for this change. Unfortunately, we could not measure food intake continuously, but the lack of a substantial body weight alteration argues against a substantial reduction in ad libitum food intake. This might suggest a context dependency (ubiquitous food availability vs. restricted feeding paradigms) similar to the discrepancies between DBS-induced alterations of ad libitum food intake compared to binge-eating behaviors^12,13,17,18^. Overall, the chronic stimulation did not substantially change body weight gain. While one might see a trend towards a slightly lower body weight or glucose concentration two hours after administration, continuous NAc DBS did not induce a substantial and sustained effect on the metabolic phenotype. One of the potential shortcomings in our study is the high-fat diet-induced obesity model. While using a ubiquitous energy-dense food source resembles an integral part of the clinical situation in obesity, it might not provide the most suitable context to investigate a procedure’s potential to “normalize” feeding behavior. Future studies should incorporate variable obesity models and different food types in order to characterize stimulation effects on feeding behavior.

We choose the stimulation parameters specified in the “Methods” section based on prior experiments performed with the predecessor rat model of our custom-made stimulator. We used a continuous 130 Hz stimulation frequency with an amplitude, pulse width and waveform that have been evaluated before in order to enable a biologically effective long-term application that did not induce tissue damage^43^. Nevertheless, a different DBS stimulation paradigm—intermittent, responsive, or at a much higher stimulation intensity might be necessary to induce the intended effects^13,31,44^.

Some more technical limitations are also present in our study design. A particularly prominent challenge is the issue of ensuring the continuity and stability of the electrical stimulation over time. We checked proper functioning every 8–10 days by reading the light sequence response indicating an intact power supply and ongoing stimulation with the intended parameters. Animals not fulfilling this criterion were excluded (n = 2). Nevertheless, we could have missed aspects of within-system malfunction, like cable or electrode breakage. Furthermore, we did not implement a non-operated control group to evaluate the effect of the surgery and the implanted device itself on the metabolic phenotype. Given the moderate obesity expression with respect to body weight gain and blood sugar elevation, a more pronounced phenotype could have increased the sensitivity and interpretability of the experiment.

An additional chow-fed “SHAM stimulation” group could have helped to determine the relative expression of the obese phenotype in the control group within the specific circumstances of our experimental design. The > 25% body weight gain over the 5 weeks stimulation period and the resulting body weight of 40.8 ± 3.23 g (Fig. 2) indicate nevertheless the presence of an obese phenotype and support the usability of the chosen experimental parameters.

In summary, our results do not promote the applicability of continuous high-frequency NAc shell DBS as approach to induce a substantial and sustained effect on the body weight development in HFD fed mice. However, DBS can alter obesity-related parameters with differing short and long-term effects. Since our data reflect only one specific stimulation paradigm used in a singular obesity model, the results do not allow evaluating DBS in general as treatment option in morbid obesity. Future research will need to employ time and context-sensitive experimental designs to assess the potential of DBS for clinical translation in this area. Our data encourage continuous stimulation paradigms to increase flexibility for comprehensive phenotyping, especially regarding neurobehavioral and metabolic effects.

## Methods

### Animals

Twenty-four 10 to 12 weeks old C57BL/6J mice were individually housed on a 12 h light/dark schedule (light on at 6:00 AM) at the center for cardiovascular research, Charité University Medicine Berlin, Germany. Following previously published protocols, animals were given a high-fat diet and tap water ad libitum to induce weight gain^45^. The high-fat diet contained 60% kJ from fat (D12492, Research Diets, New Brunswick, NJ). Animals were housed individually. The high-fat diet started 2 weeks before the microstimulator implantation. After surgery, animals had 14 days recovery time during which the body weight reached at least the preoperative level and showed a consistent further increase before randomization and initiation of the first stimulation.

All experiments were approved in advance by the local animal welfare authority (Landesamt für Gesundheit und Soziales, Berlin, Germany, G0095-17) and conducted in compliance with the institution's ethical guidelines and in accordance with EU Directive 10/63/EU and the ARRIVE guidelines.

### Custom made stimulator

For continuous stimulation, we used a custom-made, small-scale stimulation device developed by the Movement Disorders and Neuromodulation Section, Charité University Medicine Berlin. The layout and schematic of the implantable pulse generator (IPG) was developed with EAGLE software (Version 7, Cadsoft©), and the printed circuit boards (PCBs, thickness 0.3 mm) were manufactured and assembled at BZM-Thiel electronics GmbH in Berlin, Germany. This circuit board provides small dimensions (including battery, 12.6 × 12.6 × 5.2 mm) and low weight (1.6 g). The battery power can provide continuous DBS for up to 50 days with a confidence interval of 5% to the 3 V compliance voltage at a maximum frequency of 130 Hz (96 µA cathodic current, 8 µA anodic at two outputs). In addition, this unit houses a custom-made dual-channel low-power current source for biphasic, bilateral stimulation up to 20 kΩ at two time-delayed output channels. The stimulation was delivered through a cathodic pulse first at two custom-made monopolar electrodes, which have a diameter of 150 µm and are made of iridium (pure iridium 99.9%, ADVENT Research Materials Ltd, Eynesham, England). Finally, the impulse generator board, lead wires, and electrical joints were encapsulated with a thin layer of dielectric epoxy resin to protect them from mechanical damage and exposure to body fluids. (RECKLI^®^-EP-Elektroharz, Herne, Germany).

### Surgery

After 2 weeks of housing, we performed microstimulator implantation surgery on all mice under general anesthesia induced and maintained with isoflurane inhalation (5% for induction and 2% for maintenance). The surgery protocol is as follows:

(1) The mouse was first positioned in a stereotactic frame (David Kopf Instruments CA, USA) with its head securely fixed with atraumatic ear bars. (2) Before making an incision was made on the head, a subcutaneous injection of 0.2 ml of 0.25% bupivacaine hydrochloride (Carbostesin^®^, AstraZeneca, Germany) was administered. (3) The skull was adjusted to the flat position with an alignment tool (David Kopf Instruments, CA, USA). (4) Two stainless steel screws (PlasticsOne, USA) were inserted into the back of the skull. (5) Two customized electrodes were then implanted bilaterally into the nucleus accumbens shell according to the following coordinates in reference to Bregma: AP = + 1.34 mm, ML = + 0.52 mm (left side); ML = − 0.53 mm (right side), DV = − 4.5 mm. (6). A scheme of the electrode positions and a representative histological image are depicted in the supplementary material. The electrodes were secured with acrylic dental cement (Technovit^®^, Heraeus-Kulzer, Germany). (7) The stimulator board was positioned behind the electrodes on the surface of the screws, with the battery facing down. The IPG, electrodes, wires, and screws were then mounted together with acrylic dental cement, resulting in a fixed setup weighing around 2.5 g. (8) The skin was sutured, and the mouse was allowed to recover. (9) The mouse was given metamizole (4 μL/g bodyweight in 50 ml drinking water, Novaminsulfon^®^ Zentiva, Germany) for 48 h after the surgery.

### Stimulation protocol and parameters

After recovering from surgery, animals were assigned to either the high-frequency stimulation (HFS) group or the control group (the assignment was made by baseline weight balance). Stimulation parameters were set at 130 Hz, 96 µA of constant current, and a pulse width of 60 µs, using a cathodic square-wave pulse followed by an anodic pulse with a peak of 8 µA for 655 µs. We started with intermittent stimulation. During the initial behavioral assessment and metabolic phenotyping, we compared sessions with the stimulator activated and deactivated for each animal in the HFS group to evaluate acute DBS effects. After that, HFS mice received continuous stimulation for 5 weeks, with only minimal handling for body weight measurements every 3 days. The stimulation was switched on and off using a remote control that communicated with the IPG via electromagnetic waves. To activate the stimulator, we took the individual animal out of the home cage and put it on the grid of an empty cage. A slight pull was applied to the tail to ensure a straight body posture. We positioned the remote control in close proximity to head (< 2 mm, no direct contact needed). After 3–5 s readout and reprogramming was completed. Successful activation was indicated by a specific light sequence, confirming the device's proper functioning. We repeated this procedure at weeks two and five to confirm persistent active stimulation. The sham group remained unstimulated during the whole experiment. We handled animals in a parallel fashion to prevent potential stress-related effects from affecting HFS and sham treatment outcomes.

### Activity and calorimetry analysis

Each mouse was placed in a separate metabolic cage (Lab Master, TSE system, Germany), where their food intake, energy expenditure, and locomotor activity were automatically monitored and recorded. Each recording session lasted 7 days (3 days for adaptation and 4 days for active measurements). In the first session, we evaluated acute stimulation effects. We activated the stimulator at day 4 for 24 h, and then switched off at day 5 for 24 h. We reactivated at day 6 and paused again for day 7. A second recording session was performed at the end of the 5 weeks of chronic stimulation, without altering stimulation parameters for the HFS group. During this second measurement, the stimulator was not switched off at any point and the recording went on uninterrupted for the complete session. The aim was to compare the HFS group with the sham group for any differences resulting from the cumulative effect.

### ipGTT

After the chronic stimulation phase, ipGTT was performed following a standard protocol. Animals were fasted overnight for 12 h. Tail tips were cut off to obtain blood for glucose measurements. Glucose was injected intraperitoneally at a dose of 2 mg/g body weight. Blood glucose was measured at 0-, 30-, 60- and 120-min time points. Finally, the mean blood glucose levels of the two groups were compared.

### Behavior tests

All mice underwent three rounds of social interaction and open-field testing: a baseline test with stimulation off, a behavior assessment during the acute stimulation phase, and a behavior assessment after the chronic stimulation phase. All tests were conducted in a sound-attenuated room during the light-on hours between 12:00 noon and 6:00 PM.

#### Open-field test

We evaluated spontaneous locomotor activity (average velocity, total distance traveled) and thigmotaxis of the mice using a video-tracking system (Biobserve Viewer) monitoring a white open-field arena (50 × 50 × 50 cm). The test was started by placing the mouse from its home cage to the center of the open-field arena. We measured the mouse’s overall locomotor activity over a 30-min period and calculated the thigmotactic ratio to assess thigmotaxis. The thigmotactic ratio represents the proportion of distance traveled close to the walls (< 2.5 cm) compared to the total distance traveled during the initial three-minute exploration^39^.

#### Social interaction test

We put the mouse into an open-field arena (50 cm × 50 cm × 50 cm) that contained an empty, transparent Plexiglas container centered against one arena wall. After 150 s, the mouse was removed and the container was replaced with one harboring another mouse. Afterwards, we re-introduced the first mouse into the open-field arena for another 150 s. Animal position and movement were continuously video-monitored. The social interaction ratio (SI ratio) was calculated by dividing the time the mouse spent in the interaction zone (a 16 × 28 cm area in close proximity to the other mouse in the container) in the presence of the second mouse, by the time spent in the interaction zone when the second mouse was absent^46^.

### Histology and electrode placement verification

Each mouse was sacrificed with isoflurane overdose, followed by perfusing with 4% paraformaldehyde in phosphate-buffered saline. The stimulator was then removed. The brain was taken out intact and frozen at − 80 °C. Next, all brains were cut into 40 µm-thick coronal slices using a cryostat (Leica, Germany) and subjected to Nissl staining. Finally, the position of electrode tips was controlled by confirming their placement in the NAc shell region under a microscope against a mouse brain atlas^47^.

### Data analysis

All data are presented as means ± SEM. Body weight, blood glucose levels and immediate changes in energy expenditure, locomotor activity are expressed as absolute values and analyzed using Two-way ANOVA. Differences in food intake, water intake, energy expenditure, locomotor activity, social interaction ratio and thigmotactic ratio were analyzed by Student’s *t*-test (paired test for comparison within the group and acute vs. chronic stimulation, unpaired for between groups). Significant differences were defined as p < 0.05. For the main parameters food intake and body weight after chronic stimulation, no multiple comparison correction was performed. For the exploratory analysis (metabolic/behavioral changes after chronic stimulation) significance levels were adjusted for multiple comparison using Holm’s method. An automated video tracking software was used to analyze mouse behavior in open-field and social interaction tests. Statistical analysis was performed with GraphPad Prism (GraphPad Prism 9.3.0 (345) Macintosh Version by Software MacKiev © 1994–2021 GraphPad Software, LLC).

### Supplementary Information

Supplementary Information.

## Data Availability

The datasets used and/or analyzed during the current study are available from the corresponding author on reasonable request.
