# Supplementary material for: Continuous deep brain stimulation of the nucleus accumbens reduces food intake but does not affect body weight in mice fed a high-fat diet
Source: Sci Rep. 2023 Nov 2;13:18952. doi: 10.1038/s41598-023-45511-7 (PMC10622429; doi:10.1038/s41598-023-45511-7)
Supplement: Supplementary file 1 — Supplementary Information. [file 41598_2023_45511_MOESM1_ESM.pdf]

Continuous deep brain stimulation of the nucleus accumbens reduces food intake but does not prevent diet-induced obesity in mice.

Hounchonou F.H.[1,2,4], Tang Hui[1,2], Paulat Raik[3], Kühn Andrea[3], Spranger Joachim[1,2], van Riesen Christoph[3,5], Maurer Lukas[1,2,*]

Supplementary material

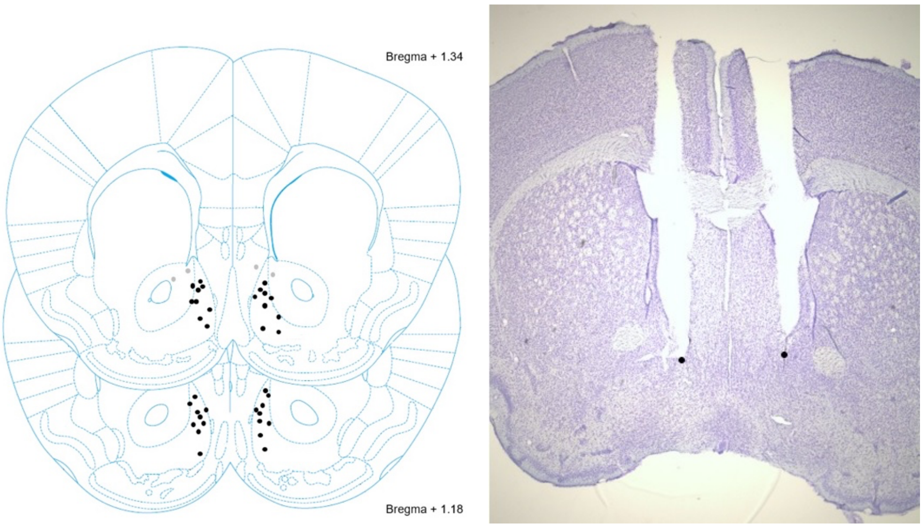

Electrode location: The black points on the right picture show the top of the electrode in the Nac Shell according to the mouse brain atlas (Paxinos, G. & Franklin, K. B. Paxinos and Franklin's the mouse brain in stereotaxic coordinates. Academic press, 2019).
